# Supplementary material for: Estimating the asymptomatic proportion of SARS-CoV-2 infection in the general population: Analysis of nationwide serosurvey data in the Netherlands
Source: Eur J Epidemiol. 2021 Jun 10;36(7):735–9. doi: 10.1007/s10654-021-00768-y (PMC8191704; doi:10.1007/s10654-021-00768-y)
Supplement: Supplementary file 1 — Supplementary file1 (DOCX 172 kb) [file 10654_2021_768_MOESM1_ESM.docx]

**SUPPLEMENTARY MATERIALS**

To accompany the article *Estimating the asymptomatic proportion of SARS-CoV-2 infection in the general population: Analysis of nationwide serosurvey data in the Netherlands*

**Methods S1: serological testing**

Serum samples were tested for the presence of IgG antibodies to SARS-CoV-2 Spike S1. Sera were diluted in SM01 buffer (Surmodics, USA) supplemented with 2% FCS, added to the beads coated with Spike S1 proteins (Cat No. 40591-VO8H, SinoBiological, China) and incubated for 45 mins at room temperature while shaking (600 rpm). Next, samples were washed three times (PBS) and PE-conjugated goat anti-human IgG was added (1/400 in PBS) and incubated for 30 mins as before. Finally, samples were washed again and acquired on a LX200 or FlexMap 3D (Luminex). Concentrations were interpolated from a reference consisting of pooled sera using a 5-parameter logistic fit.

For PICO-1, sensitivity and specificity were estimated at 84.4% and 99.0%, respectively, using ROC analysis (Vos et al., 2020; den Hartog et al., 2020). For PICO-2, sensitivity and specificity were estimated at 94.3% and 99.9%, respectively, using mixture modelling methods (Vos et al., 2021).

**References**

Vos RA, den Hartog G, Schepp RM, Kaaijk P, van Vliet J, Helm K, et al. Nationwide seroprevalence of SARS-CoV-2 and identification of risk factors in the general population of the Netherlands during the first epidemic. J Epidemiol Comm Health. 2020. <http://dx.doi.org/10.1136/jech-2020-215678>.

Den Hartog G, Rutger M, Schepp MK, Geurts van Kessel C, van Beek J, Rots N. et al. SARS-CoV-2–specific antibody detection for seroepidemiology: a multiplex analysis approach accounting for accurate seroprevalence. J Infect Dis. 2020; 222(9):1452-1461.

Vos ER, van Boven M, een Hartog G, Backer JA, Klinkenberg D, van Hagen CC. Associations between measures of social distancing and SARS-CoV-2 seropositivity: a nationwide population-based study in the Netherlands. Clin Infect Dis. 2021. https://doi.org/10.1093/cid/ciab264.

**Table S1.** Crosstabulation of age-aggregated PICO data by serostatus and symptomatic/not, for ECDC case definition (main analysis**)** and for 'any symptom' definition (sensitivity analysis 2) of symptomatic. Percentages are with respect to row totals.

|  | ECDC case definition | | Any symptom | |
| --- | --- | --- | --- | --- |
|  | Symptomatic (%) | Not symptomatic (%) | Symptomatic (%) | Not symptomatic (%) |
| Seropositive | 190 (63%) | 110 (37%) | 227 (76%) | 73 (24%) |
| Seronegative | 2048 (28%) | 5169 (72%) | 3592 (50%) | 3625 (50%) |
| Total | 2238 (30%) | 5279 (70%) | 3819 (51%) | 3698 (49%) |

**Table S2.** Model fit (according to AIC) of model variants (all models include intercept). Selected model (with lowest AIC) in **boldface**.

**Included terms AIC AIC difference***

1. Intercept only 9901.1 205.8

2. Serostatus 9812.8 117.5

3a. Serostatus + age (linear term) 9728.4 33.1

3b. Serostatus + age (P-spline) 9696.7 1.4

**4. Serostatus** × **age 9695.3** – **(separate P-splines fitted for positive & negative)**

5. Serostatus × age (separate P-splines) + sex 9696.7 1.4

6. Serostatus × age (separate P-splines) + educ 9697.2 1.9

7. Serostatus × age (separate P-splines) + 9698.7 3.4

household size category

8. Serostatus × age (separate P-splines) + 9696.1 0.4

healthcare occupation

9. Serostatus × age (separate P-splines) + round 9697.2 1.5

* Compared with selected model (4). *Round* is an indicator variable for PICO-1 vs. PICO-2.

**Table S3.** Model fits (according to AIC) for sensitivity analysis 2. Selected model (with lowest AIC) in **boldface**.

**Included terms AIC AIC difference***

1. Intercept only 12912.3 305.1

2. Serostatus 12781.1 173.9

3a. Serostatus + age (linear term) 12696.5 89.3

3b. Serostatus + age (P-spline) 12614.8 7.6

4. Serostatus × age 12613.3 6.1
 (separate P-splines fitted for positive & negative)

5. Serostatus × age (separate P-splines) + sex 12611.2 4.0

**6. Serostatus** × **age (separate P-splines) + sex + educ 12607.2** –

7. Serostatus × age (separate P-splines) + sex + educ + 12610.7 3.5

household size category

8. Serostatus × age (separate P-splines) + sex + educ + 12609.0 1.8

healthcare occupation

9. Serostatus × age (separate P-splines) + sex + educ + 12609.2 2.0

round

* Compared with selected model (6)


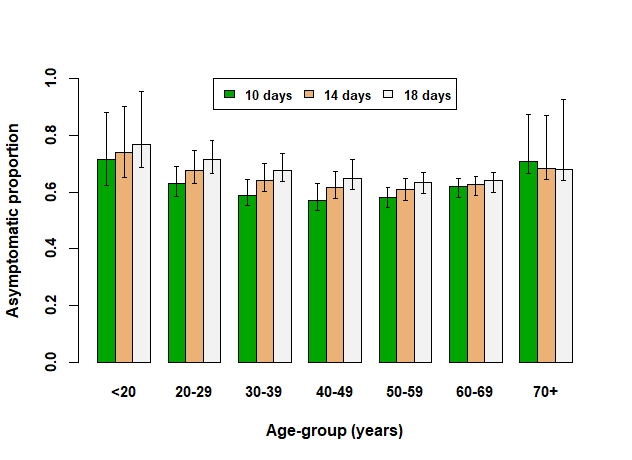
**Fig. S1.** Results of sensitivity analysis 1: estimated asymptomatic proportion per 10-year age-group comparing three different assumptions regarding the size of the antibody response window (corresponding to the median number of days assumed needed to build an IgG response).


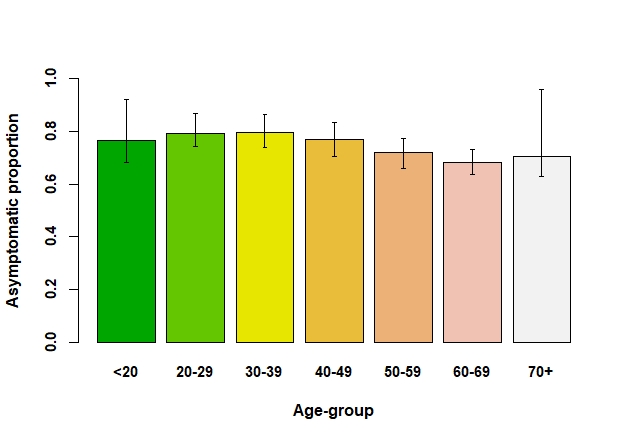
**Fig. S2.** Results of sensitivity analysis 2 ('any symptom' definition): the estimated asymptomatic proportion per 10-year age-group, as derived using the attributable risk approach.
